# Association Between Rectal Spacer Use and Erectile Dysfunction Diagnosis Among Men Receiving Prostate Radiotherapy: US County‐Level Analysis

**DOI:** 10.1002/cam4.71362

**Published:** 2025-12-13

**Authors:** Ryan Hankins, Sean P. Collins, Ryoko Sato, Parthiv Mehta, Samir Bhattacharyya, Emmanuel Ezekekwu, Daniel A. Hamstra

**Affiliations:** ^1^ Department of General Urology MedStar Georgetown University Hospital Washington DC USA; ^2^ Radiation Oncology Morsani School of Medicine, University of South Florida Tampa Florida USA; ^3^ Boston Scientific Marlborough Massachusetts USA; ^4^ UroPartners Cancer Treatment Glenview Illinois USA; ^5^ Radiation Oncology Baylor College of Medicine Houston Texas USA

## Abstract

**Background:**

Rectal spacers have been shown in clinical studies to reduce side effects of radiotherapy (RT) in prostate cancer (PCa) patients. In addition, secondary analyses also showed reduced erectile dysfunction following PCa RT with the use of a rectal spacer. However, this association with erectile dysfunction (ED) in large‐scale real‐world settings remains unexplored. This study evaluated the association between rectal spacer use and the prevalence of ED diagnosis among PCa patients receiving prostate RT at the US county level.

**Methods:**

This study utilized Medicare 5% and 100% Standard Analytic Files to analyze county‐level data. The analytical sample included adult PCa patients receiving RT—comprising stereotactic body radiation therapy (SBRT), intensity‐modulated radiation therapy (IMRT), proton beam radiation therapy, and brachytherapy—between January 2015 and March 2023. The primary outcome was the county‐level proportion of RT patients diagnosed with ED between January 2015 and March 2023. The primary explanatory variable was the proportion of patients receiving prostate RT utilizing a rectal spacer from 1 to 5 years prior to an ED diagnosis. Zero‐inflated Poisson regression models were used to assess the association between rectal spacer use and ED prevalence at the county level, controlling for county‐level PCa patient characteristics (median age and racial composition) and general population characteristics (median age, racial composition, and median household income). State‐level fixed effects accounted for regional variation. Data for general population characteristics were obtained from the 2020 Agency for Healthcare Research and Quality Social Determinants of Health Database.

**Results:**

The study included 247,250 PCa patients who underwent RT across 3132 US counties between January 2015 and March 2023. The average annual prevalence of ED among PCa patients receiving RT at the county level was 1.3%. During the study period, the proportion of patients receiving rectal spacers increased from 2.9% to 18.9%. After adjusting for confounders, counties with higher rectal spacer use 4–5 years prior had a significantly lower prevalence of ED: a 10‐percentage point increase in rectal spacer use at the county level was associated with a 7.7% relative reduction in ED prevalence after 4 years (*p* < 0.001) and an 8.4% reduction after 5 years (*p* = 0.006).

**Conclusion:**

This is the first large‐scale real‐world analysis to demonstrate an association between rectal spacer use and ED prevalence among PCa patients undergoing RT. County‐level analysis suggests that increased use of rectal spacing among PCa patients receiving RT is associated with a significantly lower prevalence of ED, with benefits emerging after a 4–5‐year time lag. These findings support the long‐term benefit of rectal spacer use in preserving sexual function in PCa patients undergoing prostate RT. Future research should evaluate the etiology of the delayed benefit observed in this study.

## 1 Introduction

Radiotherapy (RT) is a standard treatment strategy for clinically localized prostate cancer (PCa). Innovations in technology and imaging have enabled oncologists to deliver higher doses of radiation to the prostate with improved precision while minimizing damage to surrounding tissues. Despite this advancement, the rectum, bladder, penile bulb, and neurovascular bundles remain vulnerable to radiation exposure due to their anatomical proximity to the prostate. Adverse side effects, such as bowel, urinary and sexual dysfunction are common and impact patients' quality of life (QOL) and remain a significant clinical concern post‐RT [1, 2].

Rectal spacers, made of biocompatible materials, including hydrogel, hyaluronic acid, and implantable balloons, have been developed to mitigate radiation exposure to the anterior rectum [3]. Clinical trials have shown that rectal spacers are safe, tolerable, and effective in expanding the prostate–rectal distance and decreasing radiotherapy exposure across RT modalities [1, 4, 5, 6, 7]. Studies have also found that rectal spacers mitigated adverse effects on QOL in terms of satisfaction and functionality in the bowel, urinary, and sexual domains [8, 9, 10].

Sexual dysfunction is a common side effect of RT, with function slowly declining over months to years post RT [11, 12, 13, 14]. ED is independent of delivery approach occurring in 25%–65% of men who undergo RT [15, 16, 17]. Psychological and endocrine factors may also contribute to ED. RT is commonly given in conjunction with androgen deprivation therapy (ADT), which can impair libido and potency and erectile function may not be restored after treatment [18, 19]. Sexual aides are commonly prescribed following RT and are moderately effective for RT‐induced ED [14, 20]. Given its significant impact on QOL, preservation of sexual function post‐treatment is important to many men, which can affect their treatment decisions [21, 22].

To date, there have been limited clinical studies assessing the long‐term association of rectal spacing on ED and overall sexual QOL [23, 24]. Hamstra et al. [23] conducted a secondary analysis of a Phase 3 clinical trial examining sexual QOL post‐RT. The use of rectal spacer decreased dose to the penile bulb which has been previously associated with sexual dysfunction following RT. Among men with adequate baseline sexual function, those with rectal spacer were more likely to maintain erectile function than those without spacer at 37 months post RT. They also maintained a higher score in the sexual domain section of the Expanded Prostate Cancer Index Composite (EPIC) [23]. In a pooled analysis, Seymour et al. [24] assessed the impact of rectal spacer use on sexual QOL following EBRT at a median of 33 months. Based on a sample with good baseline sexual function, men who did not receive a spacer were more likely to have declines in EPIC scores on sexual function, sexual bother, and sexual summary score [24].

While these findings are promising, the clinical trial setting has certain limitations and may not fully represent the experience of real‐world populations. Large, real‐world data may help complement trial findings and provide a broader understanding of the long‐term effects of rectal spacer use on ED. Thus, this study leverages large‐scale administrative data, specifically Medicare claims, to explore the real‐world uptake of rectal spacers and their association with ED prevalence among PCa patients undergoing RT over time at the county level.

## 2 Materials and Methods

### 2.1 Data and Analytical Sample

This study used aggregated county‐level data from the Medicare Standard Analytic Files (SAFs) to examine the association between rectal spacer use among PCa patients receiving RT and the average annual prevalence of ED [25]. The Medicare 5% SAFs contain demographic and enrollment information, as well as billing claims from all health encounters regardless of the location of service among the representative 5% sample of all Medicare patients. The Medicare 100% SAFs include information for all Medicare patients but are limited to claims from hospital settings. Data from both datasets were combined for this analysis, excluding patients in the 5% Medicare who received a prostate cancer diagnosis at the hospital to avoid double counting with the Medicare 100% dataset.

County‐level characteristics of the general population data were obtained from the 2015 to 2020 Agency for Healthcare Research and Quality Social Determinants of Health (SDOH) Database [26]. The database contains information across key SDOH domains: social context; economic context; physical infrastructure; and healthcare context. Information from SDOH was merged based on the year the outcome (ED prevalence) was observed. If the outcome was observed in 2021 or later, SDOH 2020 information was used. These factors were included as potential confounders that could influence both the outcome and explanatory variables (rectal spacer use).

The study population included adult males aged ≥ 65 years diagnosed with PCa cancer between January 1, 2015 and March 31, 2023 (First quarter; Q1) who underwent RT after diagnosis. RT modalities included stereotactic body radiation therapy (SBRT), intensity‐modulated radiation therapy (IMRT), proton beam radiation therapy, and brachytherapy. Patients who received a combination of any of the radiotherapy modalities post prostate cancer diagnosis, regardless of sequence, were included. Procedure codes identifying RT modality are listed in Appendix 1. Individuals were required to have continuous Medicare enrollment for 1 year prior to RT (baseline period).

Rectal spacer use during RT was identified using Current Procedural Terminology (CPT) codes 0438T and 55874. CPT code 0438T was used prior to 2018, after which it was replaced by CPT code 55874.

### 2.2 Type of Rectal Spacer

Most patients were administered SpaceOAR Hydrogel system (Boston Scientific) rectal spacers at the time of their RT treatment. SpaceOAR was approved by the FDA in 2015 and was available for clinical use in the United States several years before other products, for example, Barrigel Rectal Spacing (Teleflex Incorporated) and BioProtect Balloon Implant System (BioProtect), which were cleared in 2022 and 2023, respectively. Given the period of analysis (2015–2023Q1), only events involving the administration of SpaceOAR generated sufficient data to track outcomes beyond 1 year.

### 2.3 Outcomes and Measures

The primary outcome was the percentage of ED diagnosis among PCa patients with RT each year during the study period, measured at the county level. ED was identified using diagnosis codes based on Pan et al. [27]. In that study, International Classification of Diseases, 9th Edition, Clinical Modification (ICD‐9‐CM) diagnosis codes were used to identify ED (607.84). The current study uses the same codes after converting the ICD‐9‐CM codes to ICD‐10‐CM codes (N52.9, N52.3, N52.35, N52.39) using conversion tables provided by the Centers for Medicare & Medicaid Services.

The primary explanatory variable was the percentage of rectal spacer use among PCa patients receiving RT at the county level in the previous 1–5 years (years *t*‐5 to *t*‐1) before ED diagnosis (year = *t*). The study hypothesized that counties with higher rectal spacer use in the prior 1–5 years would have a lower prevalence of ED among PCa patients undergoing RT.

### 2.4 Statistical Analysis

Zero‐inflated Poisson regression models were used to assess the association between rectal spacer use and ED diagnosis at the county level. The models controlled for potential confounding variables among patients at the county level (median age and percentage White patients) and in the general population (median age, % White, and median household income) that might influence treatment decisions, outcomes, and interpretations of QOL after RT. State‐level fixed effects were employed to account for regional variation.

Unlike other potential adverse events post radiotherapy such as bowel and urinary dysfunctions, evaluating the impact of radiotherapy on sexual adverse events using claims data at the individual level is challenging due to selection bias. In particular, sexual behavior depends on various factors such as the presence of partners, sexual desires from self as well as from partners. Thus, the willingness to pay for addressing sexual dysfunctions might differ by individual situation, condition, and characteristics. With claims data that do not have detailed information on individual characteristics and revealed preferences, county‐level analysis might be more appropriate where selection bias at the individual level is less of a concern, once controlling for county‐level characteristics.

All analyses were performed using the Instant Health Data (IHD) software (Panalgo, Boston, MA, USA) and Stata 18.0 (StataCorp LLC, Texas, USA).

## 3 Results

Individual‐level summary statistics are presented in Table 1. The study sample included 247,250 PCa patients residing in 3132 US counties treated with RT, with or without rectal spacers, between 2015 and 2023Q1 (Appendix 2). The mean age of patients was 72.3 years (standard deviation: 6.1) at the time of RT. The patient population was predominantly White (*n* = 203,792, 82.4%), with Black patients comprising 10.7% (*n* = 26,385). A small proportion of patients (*n* = 8.745, 3.5%) had an ED diagnosis during the baseline period of 1 year prior to RT. There were 9.6% (*n* = 23,718) of PCa patients in the sample who were diagnosed with ED at any time post‐RT during the study period. The number of PCa patients diagnosed with ED increased from 2015 (*n* = 800, 3.4% of all ED diagnoses in 2015–2023Q1) to 2022 (*n* = 4365, 18.4%). During the study period between 2015 and 2023Q1, 12.6% (*n* = 31,261) of patients had a rectal spacer inserted. The percentage uptake of rectal spacers increased each year of the study period, from 0% in 2015 (*n* = 0) to 23.0% in 2022 (*n* = 8237).

**TABLE 1:** Summary statistics of patients (*N* = 247,250).

	Mean (SD)	Median	25%	75%	
Age at RT	72.3 (6.1)	72	68	76	
	***n***	**%**			
Race					
White	203,792	82.4%			
Black	26,385	10.7%			
Other	17,073	6.9%			
	***n***	**%**			
ED diagnosis 1 year prior to RT	8745	3.5%			

ED diagnosis by year					
Year	*n*	%	% Among all patients		
2015	800	3.4%	0.3%		
2016	1505	6.3%	0.6%		
2017	2186	9.2%	0.9%		
2018	2897	12.2%	1.2%		
2019	3417	14.4%	1.4%		
2020	3418	14.4%	1.4%		
2021	4084	17.2%	1.7%		
2022	4365	18.4%	1.8%		
2023Q1	1046	4.4%	0.4%		
Total	23,718	100%	9.6%		

Spacer use by year					
Year	No Spacer		Spacer		% Spacer
	*n*	%	*n*	%	
2015	23,539	10.9%	0	0.00%	0.0%
2016	24,678	11.4%	211	0.67%	0.8%
2017	26,605	12.3%	910	2.9%	3.3%
2018	27,438	12.7%	2404	7.7%	8.1%
2019	28,580	13.2%	4896	15.7%	14.6%
2020	25,465	11.8%	5767	18.5%	18.5%
2021	26,066	12.1%	7166	22.9%	21.6%
2022	27,629	12.8%	8237	26.4%	23.0%
2023Q1	5989	2.8%	1670	5.3%	21.8%
Total	215,989	100%	31,261	100%	12.6%

Abbreviations: ED, erectile dysfunction; Q1, First quarter; RT, radiotherapy; SD, standard deviation.

Table 2 presents county‐level summary statistics. The average annual prevalence of ED diagnosis at the county level was 1.3%. The percentage of patients using rectal spacers increased at the county level over time from 2.9% (5 years prior to ED diagnosis) to 18.9% (1 year prior) and 20.9% (in the same year).

**TABLE 2:** Summary statistics at county‐level.

	Percentage
% ED	
Year = *t*	1.3%
% Spacer use among RT patients in a county	
Year = *t*	20.9%
Year = *t*‐1	18.9%
Year = *t*‐2	15.0%
Year = *t*‐3	10.6%
Year = *t*‐4	6.4%
Year = *t*‐5	2.9%

Abbreviations: ED, erectile dysfunction; RT, radiotherapy.

Categorizing the data into quartiles based on rectal spacer use at the county level, counties with higher rectal spacer adoption from 1 to 5 years prior to outcome observation tended to have lower average annual prevalence of ED diagnoses (Table 3). For example, counties in the highest quartile of rectal spacer adoption (average of 61.9%) 4 years before had the lowest average annual ED prevalence (0.88%). In contrast, counties with the highest ED prevalence (1.22%) had the lowest rectal spacer adoption (5.3%) 4 years earlier.

**TABLE 3:** Trends in spacer use and ED.

	%Spacer use at corresponding year (*t*‐1 to *t*‐5)	% ED at year = *t*
Quartile by %Spacer use at year = *t*‐1		
Zero (0)	0.0%	1.00%
Lowest	8.7%	1.33%
Lower	20.6%	1.36%
Higher	39.5%	1.49%
Highest	81.8%	1.51%
Quartile by %Spacer use at year = *t*‐2		
Zero (0)	0.0%	1.01%
Lowest	7.5%	1.36%
Lower	19.4%	1.39%
Higher	34.7%	1.31%
Highest	69.3%	1.39%
Quartile by %Spacer use at year = *t*‐3		
Zero (0)	0.0%	1.00%
Lowest	6.5%	1.31%
Lower	16.5%	1.37%
Higher	30.8%	1.32%
Highest	66.9%	1.15%
Quartile by %Spacer use at year = *t*‐4		
Zero (0)	0.0%	1.05%
Lowest	5.3%	1.22%
Lower	14.8%	1.08%
Higher	28.4%	1.07%
Highest	61.9%	0.88%
Quartile by %Spacer use at year = *t*‐5		
Zero (0)	0.0%	1.06%
Lowest	4.0%	1.11%
Lower	10.8%	0.90%
Higher	23.8%	0.90%
Highest	61.6%	0.92%

*Note:* Quartile (lowest‐highest) excluding counties with 0% spacer use.

Abbreviation: ED, erectile dysfunction.

Table 4 displays regression examining the association between rectal spacer uptake in prior years and the average annual ED prevalence. After adjusting for confounders, counties with higher rectal spacer use 4 and 5 years prior had significantly lower ED prevalence: A 100‐percentage points increase in spacer use at the county was associated with a 55.0% and 58.3% reduction in the prevalence of ED diagnosis at 4 and 5 years prior, respectively (incidence rate ratio (IRR) at 4 years: 0.450, 95% confidence interval (CI) = [0.312, 0.650], *p* < 0.001; IRR at 5 years: 0.417, 95% CI = [0.224, 0.775], *p* = 0.006). While the effects of higher rectal spacer use at 1 to 3 years prior were not significant, a decrease in ED diagnosis also occurred after 2 and 3 years: a 14.2% and 19.1% reduction respectively in ED diagnosis per 100 percentage points increase in spacer use (IRR at 2 years: 0.858, 95% CI = [0.705, 1.044], *p* = 0.126; IRR at 3 years: 0.809, 95% CI = [0.632, 1.034], *p* = 0.091). These results remained consistent when the analysis was restricted to patients without a baseline ED diagnosis (Table 5).

**TABLE 4:** Association between percentage rectal spacer use in the past and % ED diagnosis at present.

	% ED diagnosis at year = *t* (IRR)				
	(1)	(2)	(3)	(4)	(5)
%Spacer adoption at year = *t*	1.067 [0.913, 1.247]	1.129 [0.967, 1.319]	1.089 [0.941, 1.260]	1.086 [0.938, 1.258]	1.054 [0.912, 1.219]
%Spacer adoption at year = *t*‐1	1.187 [0.981, 1.437]				
%Spacer adoption at year = *t*‐2		0.858 [0.705, 1.044]			
%Spacer adoption at year = *t*‐3			0.809 [0.632, 1.034]		
%Spacer adoption at year = *t*‐4				0.450*** [0.312, 0.650]	
%Spacer adoption at year = *t*‐5					0.417*** [0.224, 0.775]
*N*	8527	8467	8430	8283	8165

*Note:* Based on zero‐inflated Poisson regression with controls: patient characteristics at the county‐level (median age and percentage white of patients) and characteristics of the general population (median age, % white, and median household income).

Abbreviations: ED, erectile dysfunction; IRR, incidence rate ratio.

*** Statistically significant at < 0.01.

**TABLE 5:** Association between percentage rectal space use in the past and %ED diagnosis at present, restricting sample to RT patients with no prior ED diagnosis.

	% ED diagnosis at year = *t* (IRR)				
	(1)	(2)	(3)	(4)	(5)
%Spacer adoption at year = *t*	1.066 [0.901,1.263]	1.131 [0.957,1.336]	1.098 [0.939,1.283]	1.099 [0.940,1.284]	1.058 [0.907,1.234]
%Spacer adoption at year = *t*‐1	1.178 [0.967,1.435]				
%Spacer adoption at year = *t*‐2		0.882 [0.718,1.083]			
%Spacer adoption at year = *t*‐3			0.892 [0.687,1.160]		
%Spacer adoption at year = *t*‐4				0.497*** [0.338,0.733]	
%Spacer adoption at year = *t*‐5					0.452** [0.236,0.865]
*N*	8431	8374	8339	8192	8068

*Note:* The sample included in this county‐level analysis consists of PCa patients undergoing RT, without prior ED diagnosis (up to 1 year prior to RT). Based on zero‐inflated Poisson regression with controls: patients characteristics at the county‐level (median age and percentage white of patients) and characteristics of the general population (median age, % white, and median household income).

Abbreviations: ED, erectile dysfunction; IRR, incidence rate ratio.

*** Statistically significant at < 0.01.

** Statistically significant at < 0.05.

**FIGURE 1:**
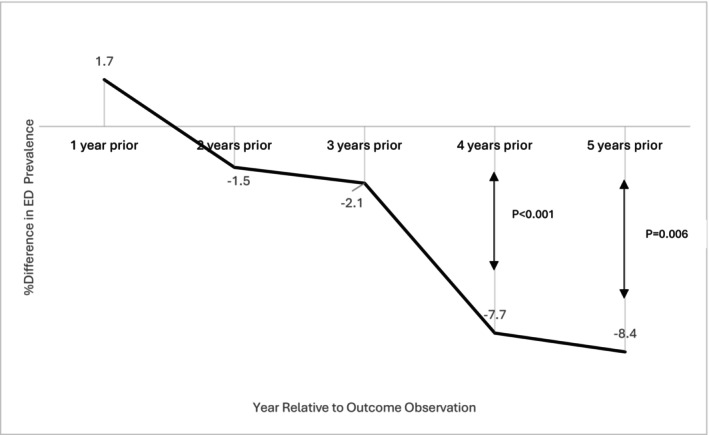
Association between percentage rectal space use (10 percentage points increase) and percentage ED diagnosis. Based on zero‐inflated Poisson regression adjusted for the following covariates: Patient characteristics at the county‐level (median age and % white patients) and characteristics of the general population (median age, % white, and median household income). ED, erectile dysfunction.

Figure 1 illustrates the effect size of rectal spacer use on ED prevalence based on the regression, as highlighted in Table 4. A 10‐percentage point increase in rectal spacer use at the county level was associated with a 7.7% reduction in ED prevalence after 4 years (*p* < 0.001) and an 8.4% reduction after 5 years (*p* = 0.006). Smaller, non‐significant reductions in ED prevalence were apparent after 2 and 3 years (1.5%, 2.1%, respectively).

## 4 Discussion

This is the first study to use real‐world, county‐level data to assess the association between rectal spacer use in the past (up to 5 years prior) and ED diagnosis in the present among PCa patients undergoing RT. It complements findings from clinical trials that reported maintained sexual QOL measures among patients with rectal spacers at 2–3 years post‐RT, contributing to an emerging evidence base that supports long‐term benefits of rectal spacers in preserving sexual function [23, 24].

In the study by Seymour et al. looking at 128 potent men treated with RT for prostate cancer, the 2/3rds of men with rectal spacer had substantially less decline in sexual function over the first 5 years as compared to the 1/3rd of men treated with RT without rectal spacer. The odds ratio for a detectable decline in sexual function based upon the EPIC QOL instrument of 3.5 (95% CI: 1.1–11.2) strongly indicated a substantially greater risk of decline in sexual function in those treated with RT without a rectal spacer which was also true for those with a larger decline that was twice the minimal clinical difference, odds ratio 3.3 (95% CI: 1.2–9.3). At last follow‐up those treated with RT alone who had erections sufficient for intercourse at baseline had a 36% chance of retaining this level of function while for those treated with RT and rectal spacer it was almost twice as great at 66% (*p* < 0.01).

To expand upon this further, the present study found a significant relation at the county level between rectal spacer use 4 and 5 years prior and reduced annual ED prevalence among PCa patients. Specifically, a 10‐percentage point increase in rectal spacer use at the county level was associated with a 7.7% reduction in ED diagnosis after 4 years, and an 8.4% reduction after 5 years. In a hypothetical scenario involving 300,000 prostate cancer patients undergoing radiotherapy in the US within a given year, with a 20% prevalence of ED post‐treatment, increasing spacer use from 2.9% to 12.9% (a 10‐percentage point increase) is estimated to reduce ED prevalence to 18.3% (an 8.4% reduction) after 5 years, benefiting approximately 5000 patients. These results highlight the potential of rectal spacers to mitigate long‐term ED complications in PCa patients treated with RT in real‐world settings.

While the findings suggest significant advantages to rectal spacer use at 4 and 5 years post‐treatment, the study does not address why a lag period exists between spacer use and less prevalence of ED. Typically, as patients age physiologically following treatment, erectile function declines in both groups (with and without spacer use). Underlying mechanisms driving this 4–5‐year time lag should be explored in future research for optimal treatment strategies.

Regarding the treatment landscape, rectal spacer uptake increased significantly among PCa patients over the study period, reflecting growing physician and patient awareness of the device and its integration into treatment protocols. At the county level, the percentage uptake of spacers increased from 2.9% 5 years prior (*t*‐5) to 20.9% at the present time (*t*). This trend was likely influenced by the introduction of additional spacer products, with two new devices entering the market between 2022 and 2023, further expanding availability and usage. These shifts underscore the growing adoption of rectal spacers as a key component of prostate cancer treatment [28].

The strengths of this analysis include the use of large national datasets comprising over 245,000 PCa patients who underwent RT. Unlike previous studies, which primarily relied on controlled clinical trial data, this study leverages real‐world data with 5 years of follow‐up. This approach enables a more comprehensive understanding of patient outcomes following RT in a broader population.

Nevertheless, this study is subject to several limitations. The analysis did not capture ED diagnoses among patients in Medicare 100% if they received a diagnosis outside of a hospital setting, which may have contributed to the low prevalence of ED diagnosis. It is also certain that absolute ED levels before, during, and after treatment are higher than those captured by diagnosis codes. However, it is unclear whether treatment‐related effects differ even if the absolute rates are lower than what would be expected if patients completed prospective validated quality of life questionnaires that more fully capture the extent and time course of ED.

As a population‐based study, this analysis does not provide information about individual‐level relations between variables. The observational nature of claims data establishes associations but does not permit causal interpretations regarding the relationship between rectal spacer placement during RT and the prevalence of ED diagnosis 1–5 years later. The estimated effect size may have been influenced by the placebo effect in this observational analysis—if patients were informed about the potential benefit of the spacer in preserving erectile function, this awareness could have affected the likelihood of ED diagnosis.

The lack of temporal changes in county characteristics, due to the unavailability of SDOH data beyond 2020, may have introduced biases in the regression. Lack of stratification by ED severity limits the clinical interpretation of results. Additionally, the study is susceptible to confounding by other factors, although adjustments were made for county‐level variables, including household income, race, and age. The Medicare data utilized for the analysis did not include information on medication use, which may have influenced ED prevalence. The analysis is also subject to bias if spacer use is associated with the likelihood of loss to follow‐up. Furthermore, a single year of continuous enrollment, intended to maximize sample retention, may not be sufficient to accurately determine actual ED status.

The reliance on Medicare claims and enrollment data excludes patients under the age of 65, which skews the sample toward older populations. This approach has implications for the overall health and functionality of patients at the time of treatment and follow‐up, potentially contributing to the low prevalence of ED. While the average age of PCa patients with RT treatment is 66–67, the mean age in this study was 72.3, which may affect the generalizability of the study findings [29]. Furthermore, because only one spacer device was approved and in clinical use for most of the study period, the findings provide limited insight into long‐term outcomes for other products approved later (beyond 1–2 years). The study also did not account for certain differences in patient characteristics, RT modalities, or disease‐specific characteristics, which could influence erectile function outcomes post‐treatment with or without rectal spacer.

Despite these limitations, this study demonstrates that, in a large, real‐world population, the use of rectal spacing is associated with a significantly lower prevalence of ED diagnosis at the county level, with a 4–5 year time lag. These findings corroborate prior clinical evidence that rectal spacers are associated with less decline in erectile function among PCa patients following RT. They also provide a foundation for future research using large‐scale datasets to explore the long‐term effects of rectal spacer use on ED and other quality of life outcomes.

## 5 Conclusions

This large‐scale, county‐level analysis provides evidence that rectal spacer use in PCa patients undergoing RT is associated with a delayed but significant reduction in ED diagnoses. These findings suggest that rectal spacers may offer long‐term protective benefits for sexual function in real‐world clinical settings. The observed time‐lagged benefit aligns with prior clinical trial evidence, reinforcing the role of rectal spacers as a valuable intervention to reduce RT‐related sexual side effects.

As rectal spacer adoption increases and new technologies emerge, these findings highlight the importance of integrating spacers into RT protocols for PCa. Future research should investigate the mechanisms underlying the delayed onset of ED reduction and evaluate the comparative efficacy of different spacer technologies to optimize patient outcomes in diverse patient populations.

## Author Contributions

**Ryan Hankins:** investigation (equal), supervision (equal), writing – review and editing (equal). **Sean P. Collins:** investigation (equal), supervision (equal), writing – review and editing (equal). **Ryoko Sato:** conceptualization (lead), data curation (lead), formal analysis (lead), investigation (lead), methodology (lead), project administration (lead), visualization (lead), writing – original draft (lead), writing – review and editing (equal). **Parthiv Mehta:** investigation (equal), supervision (equal), writing – review and editing (equal). **Samir Bhattacharyya:** validation (equal), writing – review and editing (equal). **Emmanuel Ezekekwu:** writing – review and editing (supporting). **Daniel A. Hamstra:** investigation (equal), supervision (equal), validation (equal), writing – review and editing (equal).

## Ethics Statement

The authors have nothing to report.

## Consent

The authors have nothing to report.

## Conflicts of Interest

Authors Ryoko Sato, Samir Bhattacharyya, and Emmanuel Ezekekwu are employees of Boston Scientific, the funding source for this research; Ryan Hankins is a clinical consultant for Boston Scientific. Sean Collins is a consultant for Sumitomo Pharma. The other authors declare no conflicts of interest.

## Data Availability

The data used in this research are not available due to a data use agreement with the Center for Medicare and Medicaid Services (CMS) in alignment with their privacy and security requirements and data release policies.

## Appendix 1 CPT Code for RT Modality

**Table:**

	CPT
IMRT	77385
	77386
SBRT	77373
Brachytherapy	55875
	77770
	77771
	77772
Proton Therapy	77520
	77522
	77523
	77525

## Appendix 2 Flow Chart of Analytical Sample

**Table:**

	Medicare 100%	Medicare 5%	Total
1. Prostate cancer diagnosis (all site setting)	1,955,614	147,878	2,103,492
2. Prostate cancer diagnosis (except hospital setting)	—	120,346	
3. Radiotherapy	246,066	14,587	260,653
4. Male	246,049	14,563	260,612
5. Continuous enrollment	233,948	13,785	247,733
6. Valid county information (Final sample)	233,491	13,759	247,250
